# Mental health screening in adolescents with CFS/ME

**DOI:** 10.1007/s00787-021-01734-5

**Published:** 2021-02-08

**Authors:** Maria E. Loades, Paul Stallard, David Kessler, Esther Crawley

**Affiliations:** 1 Department of Psychology, University of Bath, Bath, BA2 7AY England, UK; 2 Bristol Medical School, University of Bristol, Bristol, UK; 3 Royal United Hospital, Bath, UK; 4 Department of Health, University of Bath, Bath, UK

Psychiatric co-morbidity in adolescents is common, with the majority of those who have depression also having at least one anxiety disorder, and many meeting the diagnostic criteria for more than one anxiety disorder [1]. In our recent paper published in this journal, we reported that approximately one in three adolescents with Chronic Fatigue Syndrome (CFS/ME) has either an anxiety disorder, or major depressive disorder, or both [2].

In clinical practice, screening questionnaires which ask about depression and anxiety symptoms, such as the Revised Children’s Anxiety and Depression Scale, RCADS [3], and the Hospital Anxiety and Depression Scale, HADS [4], are often used as part of the assessment process. However, in our paper, we reported our findings of variable discriminative validity of these questionnaires for detecting anxiety and depression separately [2]. Whilst we found sufficiently accurate threshold scores for classifying those with anxiety disorders on both the 47-item and 25-item parent and child versions of the RCADS, we could not identify a sufficiently accurate threshold score for classifying those with depression. We also could not identify sufficiently accurate threshold scores on the HADS for either anxiety or depression.

Clinicians treating children with health disorders need a simple screening mechanism to identify those with co-morbid mental health problems that will require further assessment. Using one threshold score is therefore arguably more useful than calculating two separate scores (for depression and anxiety). Given the high co-morbidity between depression and anxiety in this population, we sought to identify the threshold score for mental health problems on two commonly used screening questionnaires, the RCADS-total and the HADS-total.

## Methods

We reported our methods extensively in our paper, and therefore will summarise them here (for full details see [2]). We conducted a cross-sectional study in a clinical cohort of adolescents, aged 12–18, with a confirmed diagnosis of CFS/ME. Eligible adolescents were recruited at or following their initial assessment at a specialist paediatric CFS/ME service.

They completed the following questionnaires:

Revised Children’s Anxiety and Depression Scale (RCADS) [3] – The full form of the RCADS has 47 items, 10 of which pertain to depression and 37 to anxiety. Each item is rated on a 4-point scale. Additionally, there is a brief 25-item version (10 depression and 15 anxiety items) version. We have previously tested the depression and anxiety items of the full and brief versions of the RCADS for identifying depression and anxiety, respectively [2]. We have not previously tested the total score on the self-report RCADS for identifying co-morbid anxiety and/or depression.

Hospital Anxiety and Depression Scale (HADS) [4] – The 14-item self-report HADS has seven anxiety items and seven depression items. Each item is rated on a 4-point scale. In adults, the HADS may be better as a measure of general distress rather than one which discriminates between anxiety and depression [5].

Adolescents and parent informants were interviewed using the Kiddie Schedule for Affective Disorders and Schizophrenia [6] to identify anxiety disorders and/or major depressive disorder, henceforth referred to as ‘depression’.

We used Receiver Operating Characteristic (ROC) curve analysis to examine the diagnostic accuracy of the questionnaires for detecting mental health problems (anxiety and/or depression) specifically, the RCADS total (all 47-item version and those 25 items which make up the brief version) and HADS total. We considered an area under the curve (AUC) of > 0.70 to indicate at least moderate accuracy. In clinical settings, sensitivity is more desirable than specificity; hence, we sought optimal thresholds with a sensitivity of ≥ 0.8 and a specificity of ≥ 0.7.

## Results and discussion

A total of 164 participants, mean age 15, mostly female (70%) completed the HADS and a subsample of 89 (54%) completed the RCADS. The subsample did not differ significantly on fatigue, physical functioning, HADS total, or age (data not shown).

**Fig. 1:**
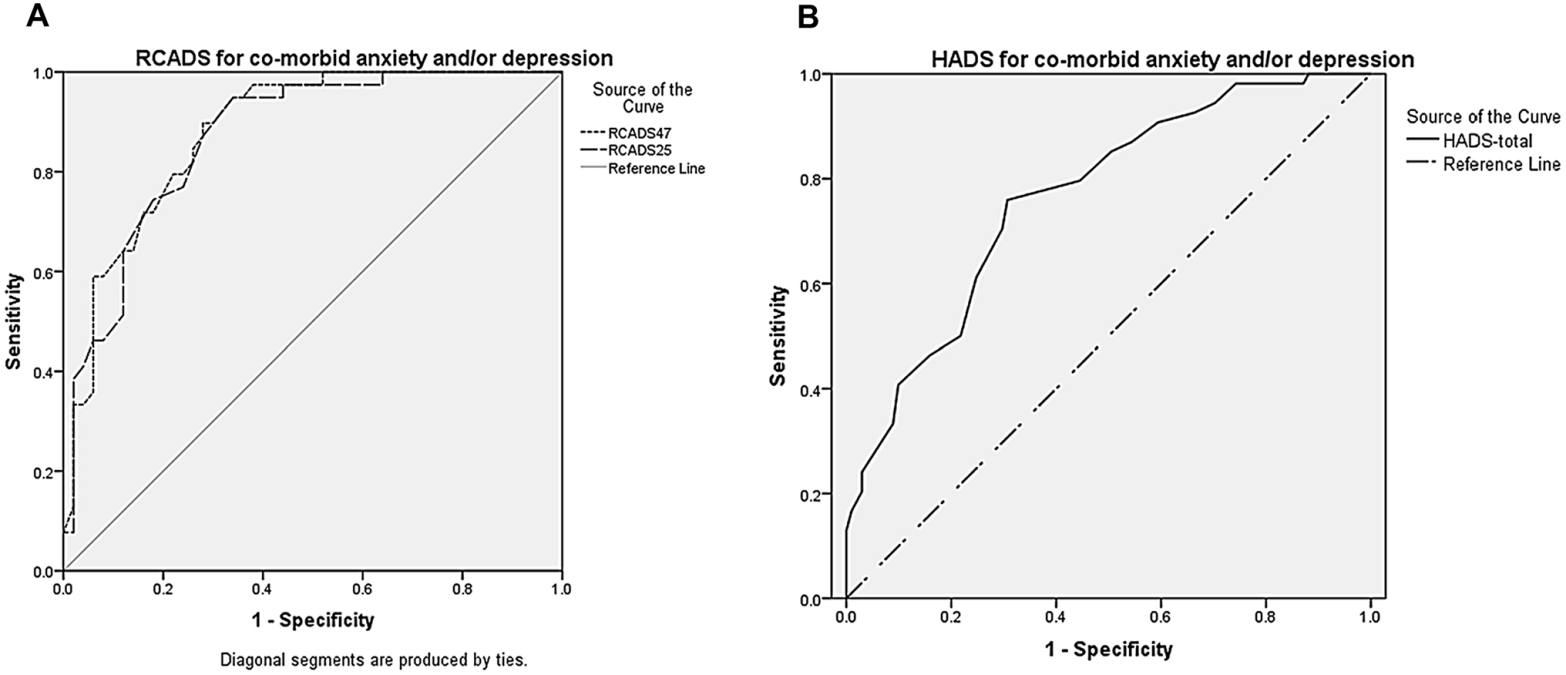
Receiver Operating Characteristic (ROC) curves

We constructed ROC curves (Fig. 1a, b). All the RCADS versions tested were at least moderately accurate for classifying co-morbid mental health problems (anxiety and/or depression)—Table 1. The HADS total was also at least moderately accurate. For classifying co-morbid mental health problems (anxiety and/or depression), we were able to identify threshold scores reaching the 0.8/0.7 requirement on the 47-item RCADS and the 25-item RCADS. We were not able to identify a threshold score reaching this requirement on the HADS, although ≥ 18 was closest at 0.76/0.69.

**Table 1:** Receiver operating characteristics for questionnaires

	Measure	AUC	S.E. of AUC	95% CI for AUC	Optimum threshold for diagnosis	Sensitivity	Specificity
Co-morbid anxiety and/or depression	RCADS-total (47 items)	0.901	0.032	0.838–0.964	** ≥ 47**	**0.879**	**0.700**
					** ≥ 48.5**	**0.879**	**0.720**
					** ≥ 49.5**	**0.872**	**0.720**
					** ≥ 51**	**0.846**	**0.740**
					** ≥ 52.5**	**0.821**	**0.740**
					≥ 53.5	0.795	0.760
	RCADS-total (25 items)	0.891	0.035	0.823–0.959	** ≥ 26.5**	0.**872**	**0.720**
					≥ 27.5	0.0769	0.740
					≥ 28.5	0.744	0.820
	HADS-total	0.765	0.039	0.689–0.842	≥ 15.5	0.852	0.495
					≥ 16.5	0.796	0.554
					≥ 17.5	0.0759	0.693
					≥ 18.5	0.704	0.703

*AUC* area under the curve; *CI* confidence interval; *HADS* Hospital Anxiety and Depression Scale *RCADS* Revised Children’s Anxiety and Depression Scale

Bold indicates that the minimum required 0.8 sensitivity/0.7 specificity criterion is met by this threshold score

For identifying co-morbid mental health problems (anxiety and/or depression), we found that both the full 47-item version and the brief 25-item version of the RCADS were sufficiently accurate and could identify threshold scores deemed to be suitably sensitive and specific to be useful for screening in a clinical setting. Given that the assessment burden of the full RCADS is almost double that of the brief version, our findings suggest that the RCADS-25 is robust to be used with this client group. A total cut-off score of ≥ 27 will correctly identify 87% of those who have co-morbid mental health problems and 72% of those who do not have a co-morbid mental health problem as diagnosed using the KSADS diagnostic interview. We could not, however, identify sufficiently sensitive and specific threshold scores on the HADS, with a score of ≥ 18 correctly identifying 76% of those who have co-morbid mental health problems and 69% of those who do not.

Participants were recruited from specialist services, so findings may not generalise to other settings, nor to those who were too severely affected to participate. The diagnostic interview was also assumed to be completely accurate, and whilst we made every attempt to ensure that it was robustly conducted, diagnostic judgements may mean that errors were made. Nonetheless, our findings suggest that a total cut-off score of ≥ 27 on the 25-item RCADS offers a simple way of identifying children with CFS/ME with co-morbid anxiety and depression.

## Data Availability

Summary data are available on reasonable request.

## Abbreviations

CFS/ME: Chronic Fatigue Syndrome/Myalgic Encephalomyelitis
DSM-5: Diagnostic and Statistical Manual of Mental Disorders version 5
KSADS: Kiddie Schedule for Affective Disorders and Schizophrenia
MDD: Major Depressive Disorder
HADS: Hospital Anxiety and Depression Scale
RCADS: Revised Children’s Anxiety and Depression Scale
